# Mechanical Properties and Microstructure of Dissimilar S355/AA6061-T6 FSW Butt Joints

**DOI:** 10.3390/ma16175950

**Published:** 2023-08-30

**Authors:** Wojciech Ziółkowski, Dariusz Boroński, Andrzej Skibicki, Radosław Stachowiak, Robert Kosturek, Lucjan Śnieżek

**Affiliations:** 1 Faculty of Mechanical Engineering, Bydgoszcz University of Science and Technology, Al. Prof. S. Kaliskiego 7, 85-796 Bydgoszcz, Poland; dariusz.boronski@pbs.edu.pl (D.B.); andrzej.skibicki@pbs.edu.pl (A.S.); radoslaw.stachowiak@pbs.edu.pl (R.S.); 2 Faculty of Mechanical Engineering, Military University of Technology, Gen. S. Kaliskiego 2 Str., 00-908 Warsaw, Poland; robert.kosturek@wat.edu.pl (R.K.); lucjan.sniezek@wat.edu.pl (L.Ś.)

**Keywords:** friction stir welding, dissimilar joints, welding parameters, mechanical properties, microstructure

## Abstract

The aim of this paper is to analyse the mechanical properties of butt joints between S355 steel and 6061-T6 aluminium alloy, as well as their relationship to changes in the structure of the material caused by welding. The effect of the tool offset was analysed in particular. For the analysis, tensile tests were carried out using macro- and mini-specimens taken from S355/AA6061-T6 joints and base materials. In addition, the macro- and microstructure of the joints was determined, the hardness profiles in the joints were analysed, and fractographic analysis of the fractures of the specimens was carried out. Based on the results of the macro- and microstructure examinations, typical friction stir welding (FSW) joint zones were characterised. The microstructure was observed in the interface line of the materials on the root side, the negative effect of which on the quality of the joint was confirmed by digital image correlation (DIC) strain analysis during the monotonic tensile test. The highest average value of *s_u_* = 141 MPa for the entire joint was obtained for a 0.4 mm tool offset. The highest average value of *s_u_* = 185 MPa for the selected joint layer was obtained for a 0.3 mm tool offset. Fracturing of the joint in the selected layer for the tool offset values of 0.3 mm and 0.4 mm occurred in the weld nugget zone (WNZ) where the lowest hardness was recorded.

## 1. Introduction

Reduction of the weight of machine and equipment components without compromising their performance is currently one of the main objectives of the industry [1,2]. The pursuit of lighter and lighter structures has an ecological and financial background because such an approach reduces the consumption of natural resources and thus production costs. One of the most common ways to reduce the weight of a machine or equipment is to use materials with the most favourable specific strength and to reduce the weight of components based on their numerical analysis. A way to reduce the weight of a device could be to use dissimilar materials within a single component. This would allow materials to be selected, e.g., based on numerical analysis, so that they locally met the required mechanical properties. The problem with such a solution is how to join dissimilar materials and the solution here may be the use of the FSW method.

Friction stir welding (FSW) was invented at The Welding Institute (TWI) in Cambridge, UK, in 1991 [3]. This method enables joining solid materials, where the parts to be joined are heated by friction and plastically deformed using a high-speed rotating tool (pin). The appropriate selection of the tool is one of the crucial elements of the process. The influence of the tool geometry on the mechanical properties and microstructure was analysed among others in the case of the AZ31B magnesium alloy [4], as well as the AA6082 T6 [5] and AA2219 aluminium alloys [6]. The authors of [7] analysed Al 1080 alloy joints made with the use of screw pitched stirrers with different pitch values. The results of their investigations showed that depending on the geometry of the tool, the tensile strength value of an FSW joint can significantly differ.

The FSW method was originally used to weld aluminium alloys, but it is now possible to join materials such as steel, titanium, nickel, or magnesium alloys [8], which has increased its popularity. 

Apart from joining identical materials, the FSW method also makes it possible to join dissimilar materials. This type of joint is characterised by different mechanical properties compared to the base materials, which creates new possibilities in terms of engineering and design. The literature contains reports on, among other things, microhardness analysis, microstructural characteristics, tensile strength, and the influence of process parameters and tool geometry on the properties of friction stir-welded joints of Al/Cu [9,10,11,12,13], Fe/Mg [14], and Al/Mg [15,16,17]. 

A particularly interesting type of dissimilar material joints made using this technology is the steel-aluminium alloy joint, which can be widely used in the industry for structural support elements. Studies available in the literature report analyses of this type of overlap joint for various configurations, e.g., AA6061/Q235 [18] and A3003/SUS304 [19], where the authors focused mainly on the assessment of the influence of process parameters on the microstructure and mechanical properties of the joint. The literature also includes scientific papers addressing the issue of dissimilar Fe/Al butt joints in configurations such as AA2024/304L [20], AA5186/mild steel [21], AA5083/SS400 [22,23], AA1050/mild steel [24], and AA6061-T6/AISI304 [25]. The main field of analysis of the above-mentioned scientific studies was the influence of process parameters (in particular, the tool rotational speed and tool offset) on the mechanical properties and microstructural characteristics of the joint, the mechanism of intermetallic compounds formation during the process, and the effect of welding in a water environment on the mechanism of microstructure evolution and grain growth. The authors of the studies [21,22] who investigated the influence of process parameters on the mechanical and macrostructural properties of steel-aluminium butt joints, presented similar conclusions on the best parameters for this type of weld. The studies demonstrated that the offset of the tool shoulder generating line towards the steel by the distance from 0.2 mm to 0.4 mm allows obtaining the relatively best tensile strength of the joint. Further increase in this parameter results in an increase in the number of steel pieces in the mixing zone, which increases the number of joint defects and decreases the strength of the entire joint. 

Although scientists and industry leaders are interested in joints of dissimilar materials, there is still a lack of conclusive study results in the field of technological parameters as well as the mechanical properties of joints. This applies in particular to steel-aluminium alloy joints that are difficult to produce and are sensitive to the accuracy of the welding process. The practical application of such types of joints in critical objects, such as load-carrying structures of vehicles, requires identification of their static and fatigue properties as well as resistance to cracking. Only a few study results of butt joint construction materials used in such applications, such as structural alloy steels and precipitation-hardened aluminium alloys, have been found in the literature. No studies devoted to the butt joint of S355 steel and AA6061-T6 aluminium alloy made by FSW have been found in the literature. These materials are very popular in the industry and this type of joint may have great applicability for example, in order to minimize a vehicle’s weight. The aim of the studies discussed in the paper is to analyse mechanical properties of the butt joint between S355 steel and 6061-T6 aluminium alloy, which are widely used materials in machine load-bearing structures and their relationship to changes in the structure of the material caused by the welding process. The effect of the tool shoulder offset, which significantly determines the quality of the resulting joints, was analysed in particular.

## 2. Materials and Methods

The subject of the tests was a 3 mm thick butt joint made by friction stir welding (FSW) of AA6061-T6 aluminium alloy and S355 steel. Both base materials of the joint are widely used structural materials. Aluminium alloy AA6061 is a typical 6000-series-grade alloy with Mg and Si as the main alloying elements. AA6061-T6 is a heat-treated, precipitation-hardened 6061 aluminium alloy, which is one of the most popular alloys of this series. It is used in the automotive, aviation, marine, and rail industries [26,27,28,29]. It has gained popularity thanks to its light weight, high ductility, and excellent corrosion resistance [30]. Its chemical composition is specified in Table 1.

Grade S355 steel is one of the most popular structural alloys with applications in most industries. It is mainly used to build the load-bearing components of machinery and equipment. This steel is regarded as universal, characterised by high strength and ductility, fine grain size, and good machinability and weldability. Its chemical composition is presented in Table 2.

**Figure 1:**
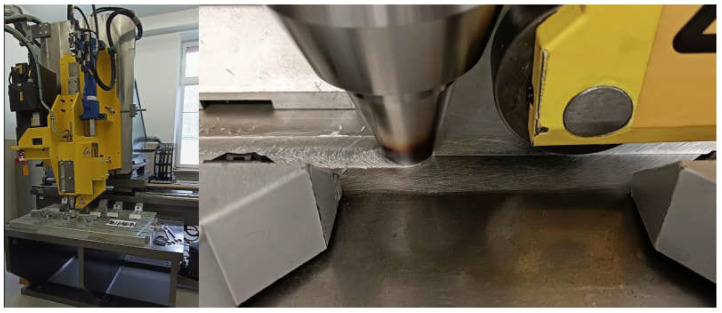
ESAB FSW LEGIO 4UT.

**Figure 2:**
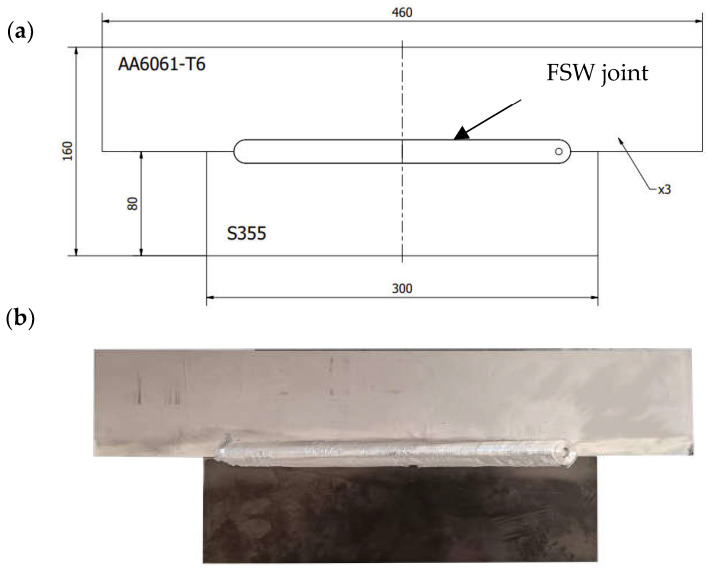
Dimensions of the welded plate (**a**); welded plate (**b**).

The research object was a 3 mm thick butt joint of plates of the AA6061-T6 aluminium alloy and S355 steel, made using the ESAB FSW LEGIO 4UT machine (Figure 1). The dimensions of the welded plates and their actual form are shown in Figure 2.

**Figure 3:**
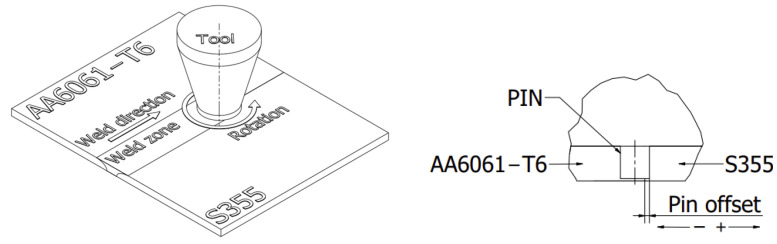
Other process parameters.

The dimension between the side surface of the tool and the side surface of the steel sheet forming the contact lines between the sheets prepared for welding is defined as the offset parameter. The interface between the sheets is the zero value of the offset parameter, while moving the tool toward the steel plate generates a positive offset value. The definition of the offset is shown schematically in Figure 3. The joints were made using three different positions of the tool offset. The offset values and other welding-process parameters used in the tests are provided in Table 3. S355 steel was welded with the AA6061-T6 aluminium alloy under conditions of tool offset control with tool penetration depth control. The welding parameters were selected based on the authors’ own experience and the results of tests carried out at other research centres for other steel grades and aluminium alloys. 

In order to analyse the mechanical properties of the joints, including the influence of the process parameters used, a number of tests were carried out, including the analysis of the micro- and macrostructure of the welded joint zone, the analysis of microhardness distribution in the joint, the analysis of strain (deformation) in the joint subjected to axial load, and the analysis of the joint strength on the macro- and microscale.

The micro- and macrostructure of the joint were examined using a metallographic microscope with magnifications of ×4, ×10, ×20, and ×40. After specimens of the joint were mounted, they were polished. The prepared polished section was subjected to etching, with the aluminium part subjected to double etching in a solution of methanol 25 mL, HCI 25 mL, HNO_3_ 25 mL, and 1 drop of HF with the etching duration of 15 s each time, to obtain images showing the macrostructure of the joint. The polished section analysed in terms of its macrostructure, was reground and repolished. Then, it was etched using a 0.5% solution of hydrofluoric acid (HF) (reagent A) and WECK reagent (reagent B) in sequences of A 45 s, B 45 s, A 45 s, B 2 × 45 s, A 45 s, and B 45 s to obtain an image of the joint microstructure. The steel part was etched with 5% Nital for 15 s.

Microhardness was measured in the joint zone using the Huatec HV-10 microhardness tester with the Vickers scale and a load of 0.3 kg. The measurements were taken every 0.5 mm in the middle of the joint cross section in a line perpendicular to the tool’s centreline. The tests were conducted in accordance with the EN ISO 6507-1 standard [31].

**Figure 4:**
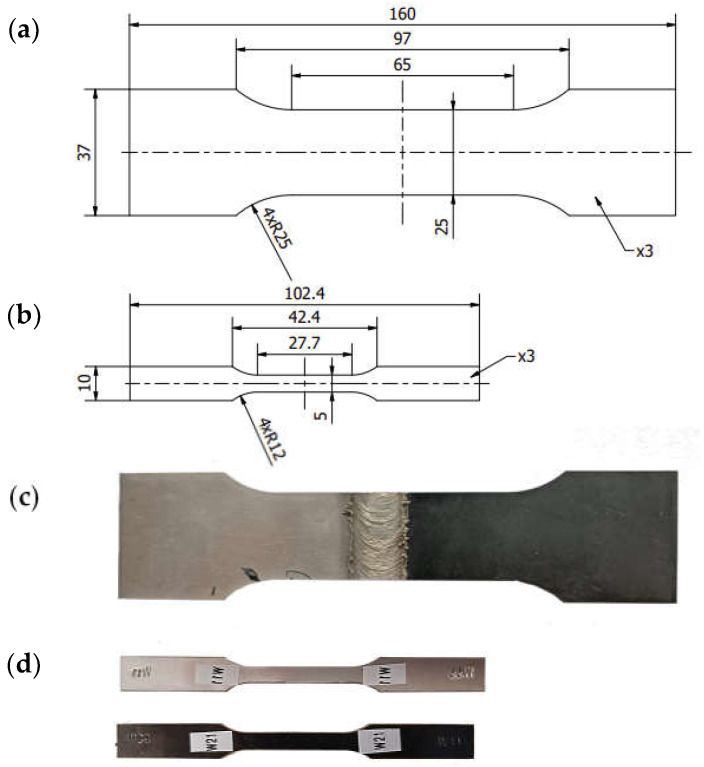
Dimensions of joint specimen W2.0 (**a**); dimensions of base material specimen W1.1 (**b**); W2.0 specimen cut from joint (**c**); W1.1 specimen cut from base materials (**d**).

The strength properties of the base materials were tested using the macrospecimens shown in Figure 4 designated as W1.1 and manufactured in accordance with the EN ISO 6892-1 standard [32]. The specimens were cut out from S355-AA6061-T6 plates by electrical discharge machining (EDM).

The strength of the joint was tested using specimens compliant with the EN ISO 4136 [33] designated as W2.0 (Figure 4) taken from 460 × 160 mm test sheets with the FSW joint made symmetrically to the longer sides. As in the previous case, the specimens were cut from the sheets by EDM.

**Figure 5:**
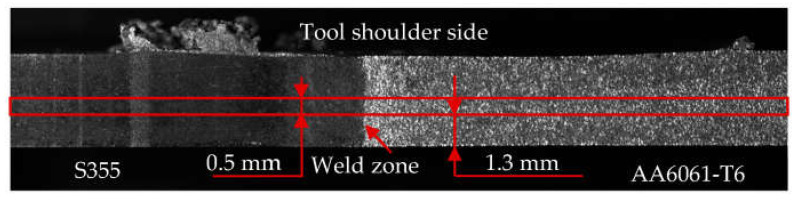
Layer arrangement.

**Figure 6:**
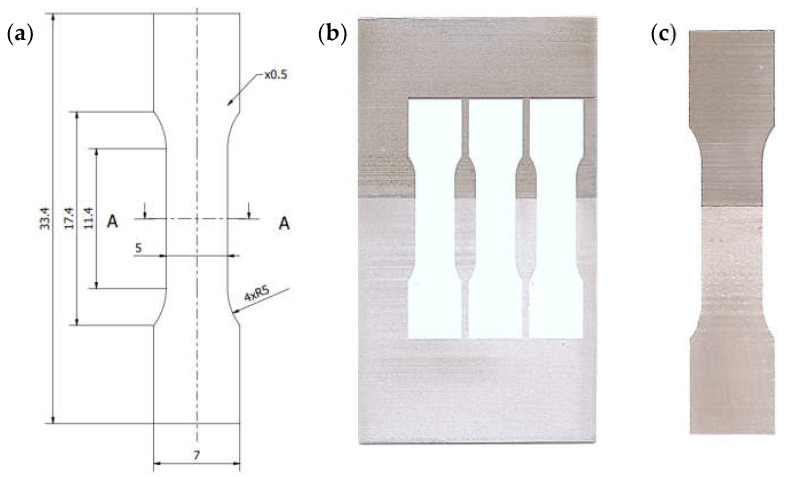
Dimensions of the test specimen WM1.0 (**a**); cut L2 layer and specimen location (**b**); WM1.0 specimen (**c**).

In addition to the tensile tests of the joints in the macroscale, tests were carried out on specimens taken from selected joint layers. Firstly, a 0.5 mm thick layer (hereinafter, referred to as L2) was cut from the S355-AA6061-T6 joint sheets by EDM as shown in Figure 5. WM1.0 specimens with a geometric form prepared according to the EN ISO 6892-1 standard [32] were cut out from the layers prepared in this way. The dimensions, location, and method of sampling are shown schematically in Figure 6.

Given the dissimilar (heterogeneous) nature of the joint, strain distribution in the joint under axial loading conditions was analysed during the strength tests using the digital image correlation method discussed later in this chapter.

Static testing of macrospecimens W1.1 and W2.0 was carried out on Instron 5966 and 8502 testing machines using extensometers characterized by measurement bases of 12.5 mm and 50 mm and using digital image correlation (DIC).

**Figure 7:**
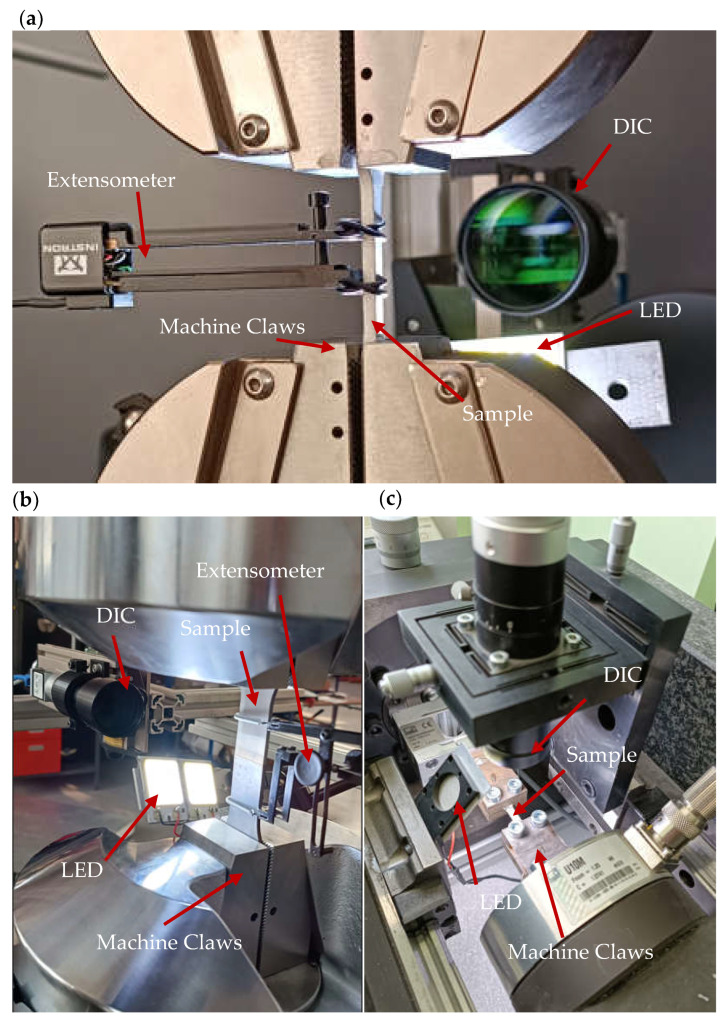
Test stand for W1.1 specimens (**a**); test stand for W2.0 specimens (**b**); test stand for WM1.0 specimens (**c**).

Micro-specimens were tested using the Micro Fatigue System (MFS) dedicated to static and fatigue testing of micro-objects [34,35]. Specimens in the MFS system are loaded using a nano- or microdrive. In the first case, a specimen is loaded using a piezoelectric actuator with a displacement resolution of 1.7 nm. The second drive system with a resolution of 1 μm is based on a precise electromechanical actuator. Given the small size of the measuring part of the specimens, the digital image correlation method [36] was used to measure the strain in the MFS system. In the DIC method, the measurement of the strain is based on the natural image of the specimen surface without additional markers applied. The image of the specimen surface is observed using VS Technology’s high-quality telecentric lenses with a micrometer-scale optical resolution and a high-resolution Basler Ace (Ahrensburg, Germany) camera. Figure 7 shows an overview of the MFS system in the measurement zone during the test.

## 3. Results and Discussion

### 3.1. Microstructure

**Figure 8:**
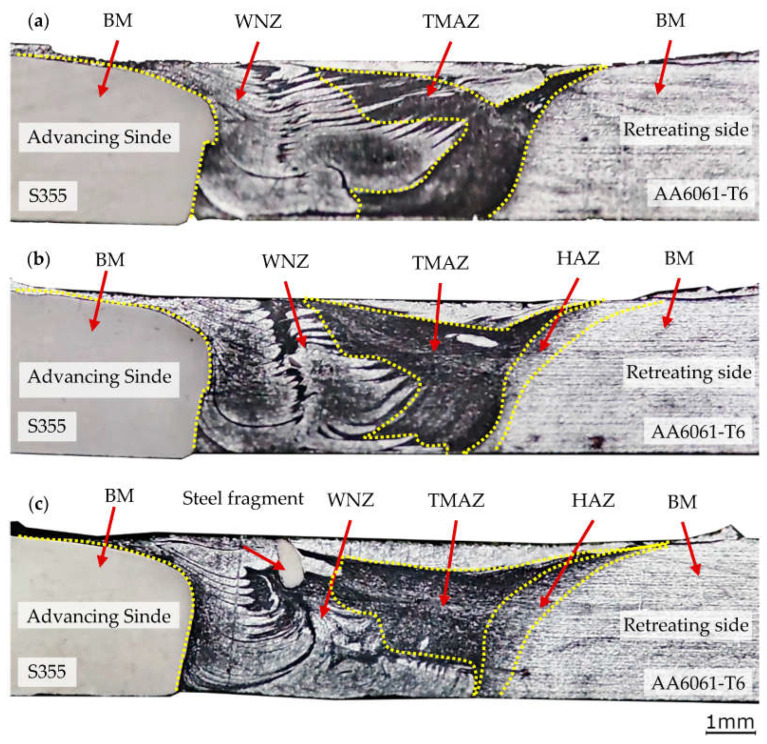
Macro-structural description of a joint made with offsets of 0.2 mm (**a**), 0.3 mm (**b**), and 0.4 mm (**c**).

The heat generated during FSW can cause significant changes in the structure of the materials being joined. This is particularly the case with precipitation-hardened alloys such as AA6061-T6. The heat introduced through friction causes, in this case, undesirable evolutions of the alloy’s microstructure, including grain growth and overaging of the strengthening phase formed in the precipitation hardening heat treatment. At the same time, the severe hot plastic deformation which occurs during the stirring of the plasticized material results in dynamic recrystallization in the WNZ forming an ultrafine grain microstructure. The refinement of grains in the WNZ partially compensates the losses in the strengthening phase in terms of the alloy’s mechanical properties. Taking into account the differences in the temperature distribution in the joint depending on the offset values used, differences in the form and size of the joint zones characteristic of the FSW method can be expected. The macrostructure of the tested joint types, as observed on the etched specimens, is shown in Figure 8, for offsets of 0.2 mm (a), 0.3 mm (b), and 0.4 mm (c), respectively. The approximate locations of the typical zones occurring in FSW joints are marked on the polished sections: the weld nugget zone (WNZ), the thermomechanically affected zone (TMAZ), and the heat-affected zone (HAZ). Moreover, the advancing and retreating sides of the joint are marked. 

The analysis of the macrostructure of joints made at different offset values reveals a different reach of the zone with a clearly visible material mixing effect (WNZ). However, unlike in the case of similar (homogeneous) materials, practically only the aluminium alloy with its structure altered by thermomechanical impacts is subject to mixing in this case. This causes the occurrence of layers with different degrees of fineness and thus different mechanical properties in the weld nugget zone. Larger offsets result in higher temperatures in the weld nugget zone and thus greater softening. This affects the shape and extent of the system of layers with a different grain size.

A more detailed analysis of the impact of the welding parameters was carried out by the comparison of the microstructure of individual joint zones.

**Figure 9:**
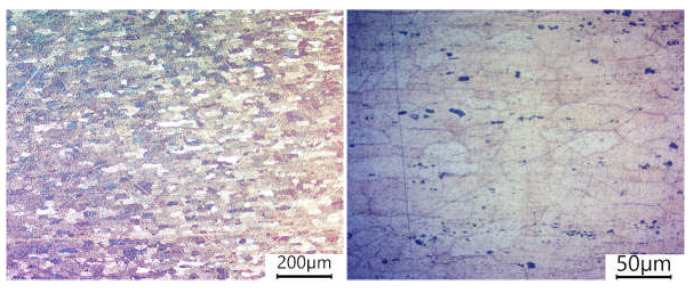
Microstructure of the AA6061-T6 base material; polarized photos.

**Figure 10:**
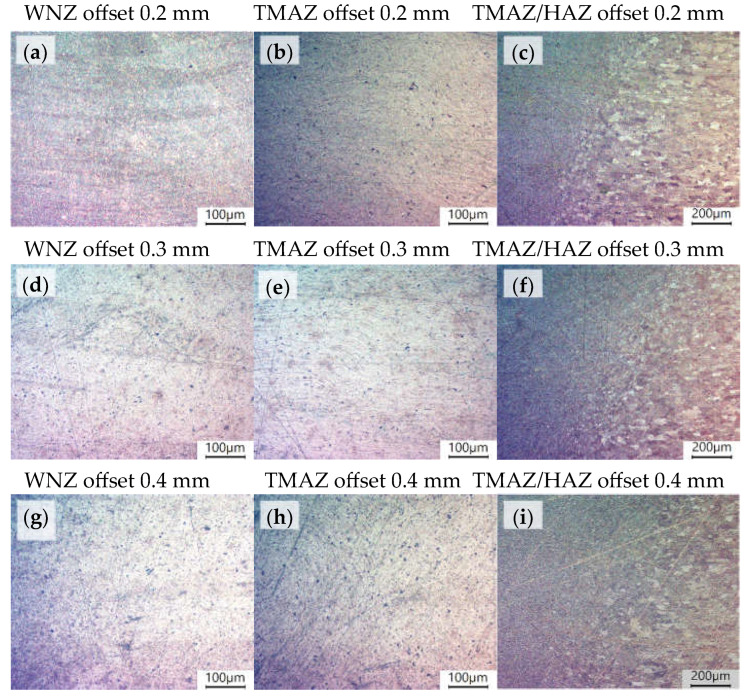
Microstructure of the S355/AA6061-T6 FSW joint, polarised photos. (**a**) WNZ offset 0.2 mm; (**b**) TMAZ offset 0.2 mm; (**c**) TMAZ/HAZ offset 0.2 mm; (**d**) WNZ offset 0.3 mm; (**e**) TMAZ offset 0.3 mm; (**f**) TMAZ/HAZ offset 0.3 mm; (**g**) WNZ offset 0.4 mm; (**h**) TMAZ offset 0.4 mm; (**i**) TMAZ/HAZ offset 0.4 mm.

The reference for the joint material microstructure is the microstructure of the base material, i.e., AA6061-T6 aluminium shown in Figure 9. The size and shape of the grain corresponds to the typical microstructure image of this material [37]. Figure 10 shows a photo of the FSW joint microstructure for each analysed value of the offset parameter. The analysis covered WNZ, TMAZ, and HAZ zones. Figure 10c,f,i show the boundary of the transition between the heat-affected zone (HAZ) and the thermomechanically affected zone (TMAZ), which was clearly visible for each offset value. It was noted that HAZ and TMAZ developed a transition boundary characterised by an intermediate grain size. This boundary varied in width for different joint types. The width increased proportionally to the offset parameter. The microstructural differences between HAZ and BM were barely discernible, among other things, due to weak thermal influences.

The TMAZ microstructure was characterised by highly elongated aluminium grains, which were arranged in stripes visible in Figure 10b,e,h, which were induced by the rotation of the pin. No differences in the TMAZ microstructure were observed between the different joint types. The WNZ mixing zone was characterised by highly refined and mixed aluminium grains. Similar observations were presented by the authors in [38]. 

**Figure 11:**
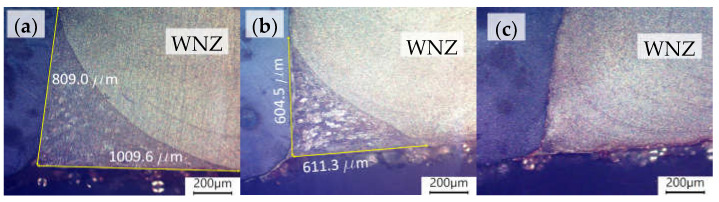
Non-welded zone of the joint for offsets of 0.2 mm (**a**), 0.3 mm (**b**) and the same zone for offset of 0.4 mm (**c**); polarised photos.

At the boundary between S355 and AA6061-T6, a grainy aluminium structure (Figure 11) resembling the HAZ/TMAZ transition boundary (Figure 10c,f,i) was observed at the joint root. This phenomenon was observed for the 0.2 mm offset (Figure 11a) and its smaller area for an offset of 0.3 mm (Figure 11b). No similar area was observed for an offset of 0.4 mm (Figure 11c). The occurrence of such structure at the boundary between a steel and aluminium alloy is indicative of the absence of the full tool impact and, therefore, of proper material bonding at this place.

**Figure 12:**
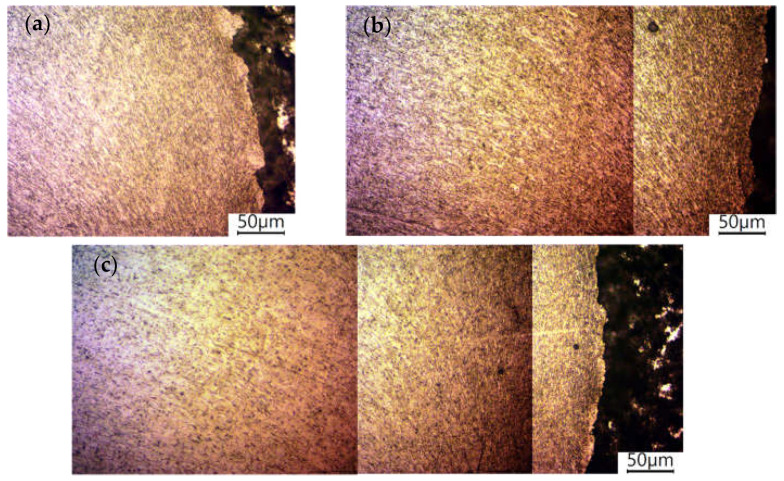
Grain size and orientation in the steel part of the joint for offsets of 0.2 mm (**a**), 0.3 mm (**b**), and 0.4 mm (**c**).

The friction stir welding process also affects the grain orientation and size of the steel part of the joint. The effect of offset on the steel part of the microstructure in the joint area is shown in Figure 12. This phenomenon is caused by the rotation of the tool’s shoulder and the impact of the temperature generated by friction. Considering the macrostructure of the joint, one can define this area as the thermomechanically affected zone in the steel part (TMAZ).

### 3.2. Microhardness

**Figure 13:**
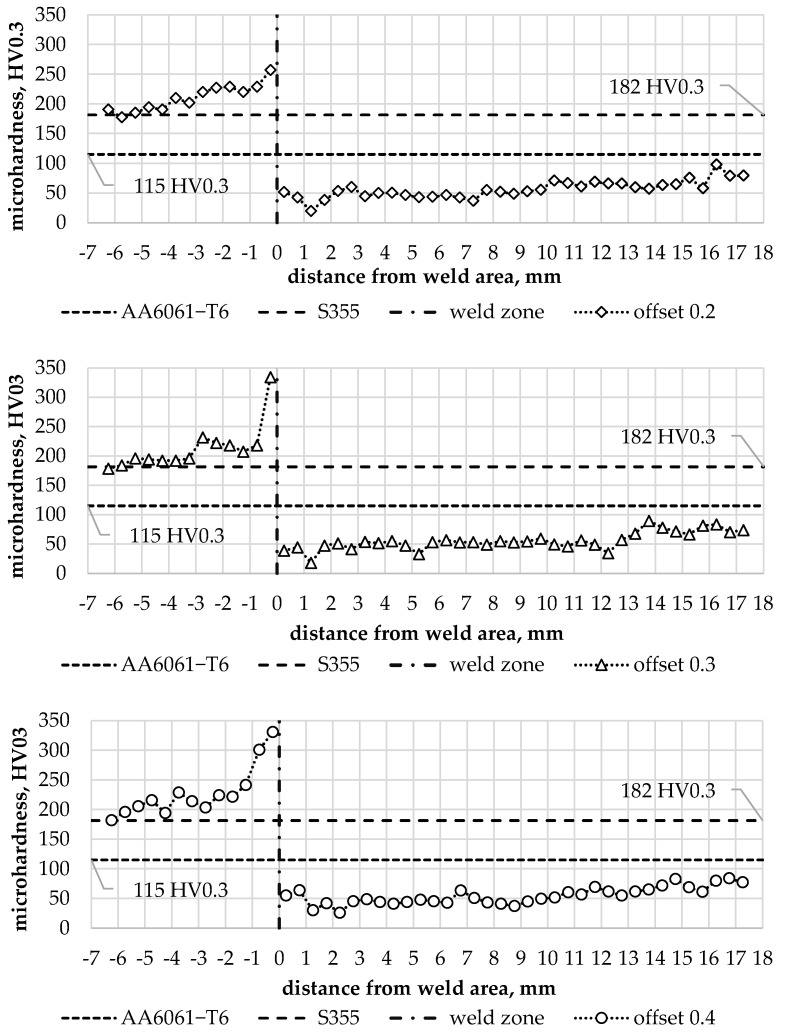
Microhardness for individual types of FSW joints analysed.

The diversity of the microstructure in the joint zone was obviously reflected in the local microhardness distribution in the joints. The microhardness distribution in the joint was determined in the transverse direction to the weld line. The microhardness profiles determined for individual S355/AA6061-T6 joint types are shown in Figure 13. The averaged microhardness of the base materials is additionally provided in the graphs.

**Figure 14:**
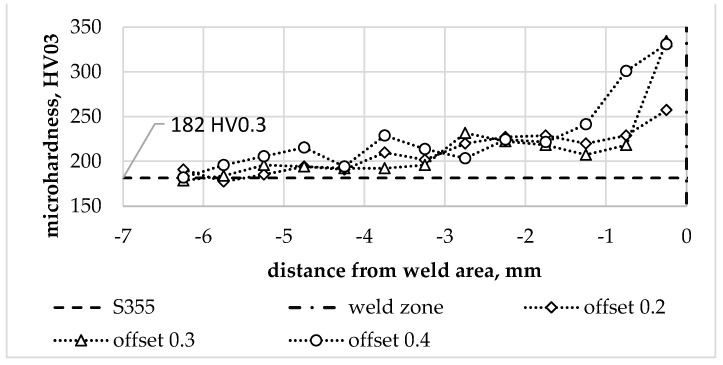
Hardness profile of the steel part for individual offset values.

The hardness of the steel near the weld line increases proportionally to the offset value. An offset of 0.2 mm increased the hardness of the steel by 42% relative to the hardness of the base material. An offset of 0.3 mm caused an 84% increase in hardness relative to the hardness of the base material over a similar distance as the 0.2 mm offset. An offset of 0.4 mm resulted in an increase in hardness of 82% relative to the hardness of the base material, although the extent of the effect was significantly greater compared to previous values of the parameter (Figure 14). The increase in hardness results from the reduction and deformation of the grain size in TMAZ on the steel side as shown in Figure 12.

**Figure 15:**
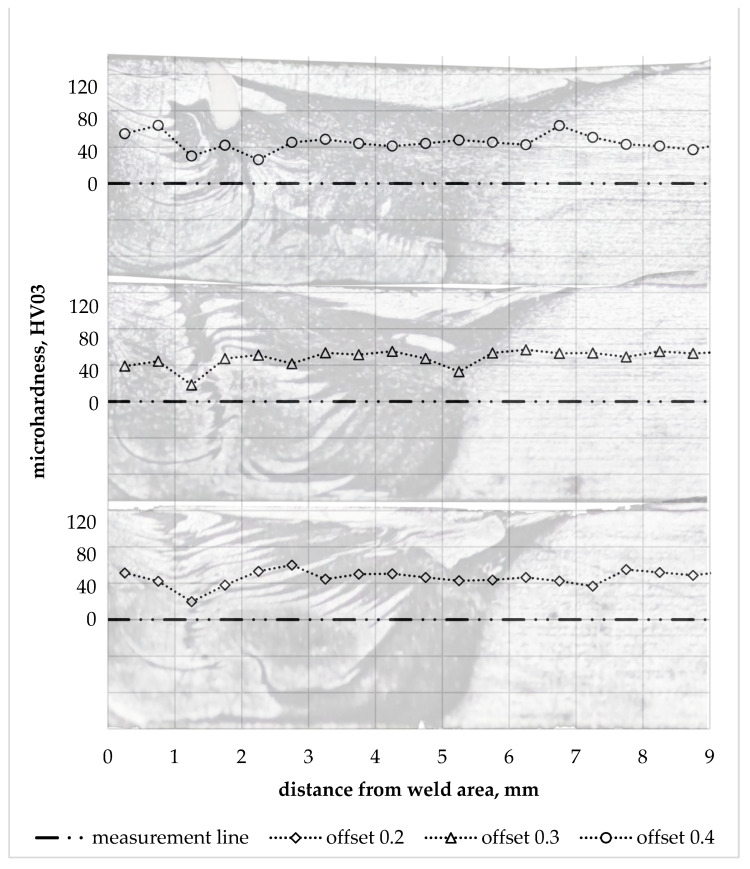
Hardness profile in the aluminium part for individual offset values.

The hardness of the aluminium in the joint zone was noticeably lower in relation to the hardness of the base material. The hardness profiles on the aluminium side for individual offset parameter values are shown in Figure 15. They show up to a 3-fold local decrease in hardness, which corresponds to the data available in the literature on the hardness differences of AA6061 alloy in the T6 and O condition [39]. The lowest hardness value was observed in WNZ for each of the offset values analysed. The decrease in hardness in this zone confirms that significant grain-size reduction cannot compensate for the effects of the aluminium alloy structure’s recrystallisation under friction and the effects of a tool-induced plastic deformation.

### 3.3. Tensile Strength

**Figure 16:**
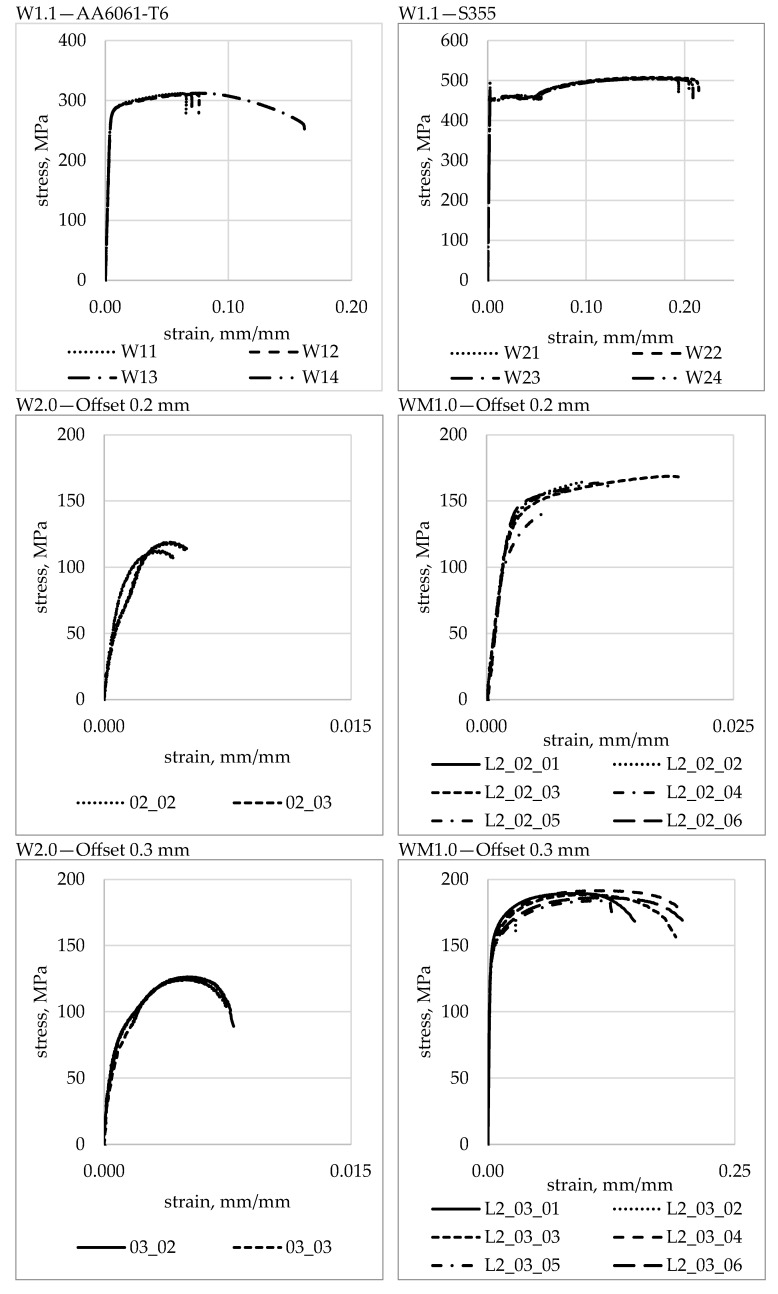

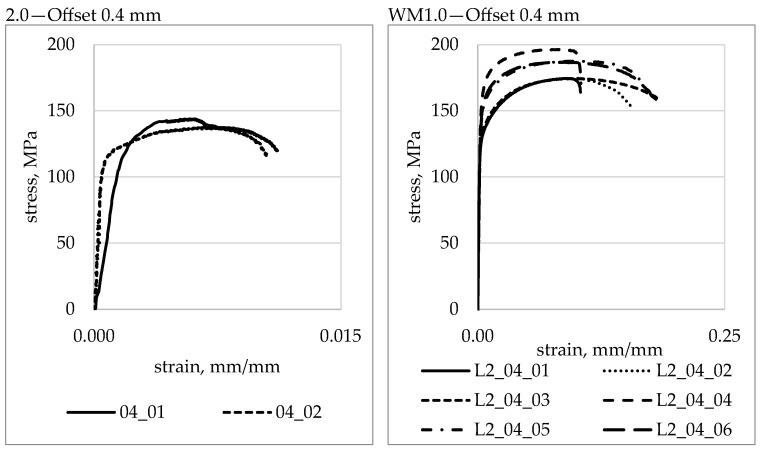
Stress-strain curves for base materials and the analysed FSW joint types.

Stress-strain curves for base materials and W2.0 and WM1.0 specimens obtained from the analysed FSW joint types are shown in Figure 16.

Table 4 shows average tensile strength values together with standard deviations for specimens W1.1 and W2.0. The average tensile strength of the entire joint (specimen W2.0) has increased proportionally to the offset parameter value and accounted for 37% for a 0.2 mm offset, 39% for a 0.3 mm offset and 45% for a 0.4 mm offset of the average tensile strength of the AA6061-T6 base material, respectively.

Table 5 presents the average values of tensile strength, yield strength, and Young’s moduli for W1.1 and WM1.0 specimens taken from layer L2. Young’s moduli for the WM1.0 specimens were calculated from the strains determined using DIC for the aluminium part of the specimens only. The average values of Young’s modulus in the layer L2 for offsets of 0.3 mm and 0.4 mm were comparable and were approximately 8% lower compared to Young’s average modulus value of the AA6061-T6 base material. The average value of Young’s modulus for a 0.2 mm offset was the lowest and was approximately 11% lower than for the base aluminium alloy.

**Figure 17:**
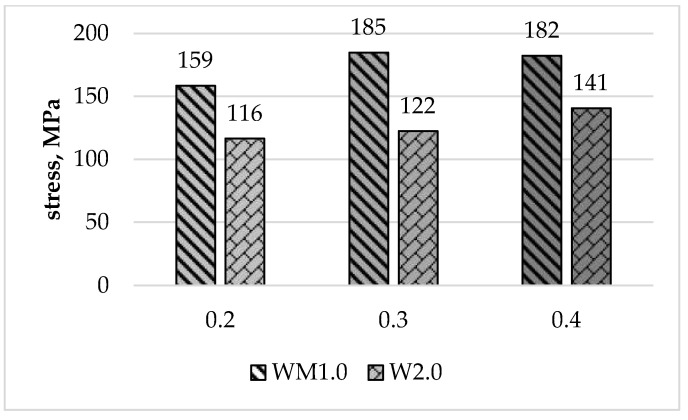
Average tensile strength of the entire joint (W2.0) and the L2 layer (WM1.0).

Figure 17 shows the average tensile strength values of the entire joint and the joint in the L2 layer for all the offset parameter values analysed. The average tensile strength values for the entire joint were lower than the tensile strength in the L2 layer for each offset value. For an offset of 0.2 mm, it was 27% lower, while for an offset of 0.3 mm, this difference amounted to 34%. The lowest difference between the tensile strength of the entire joint and the tensile strength of the joint in the L2 layer was observed for an offset of 0.4 mm, and it was 23%.

**Figure 18:**
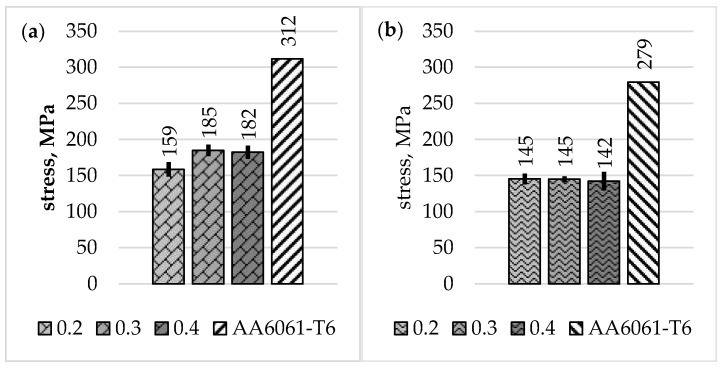
Average values of R_m_ (**a**) and R_P0.2_ (**b**) for individual offset values and AA6061-T6.

The tensile strength for an offset of 0.2 mm in the L2 layer was 51% of the tensile strength of the base aluminium and, similarly to specimens from the entire joint, was the lowest of all the parameters analysed. For the FSW joint made with offsets of 0.3 mm and 0.4 mm, it was 59% and 58% of the tensile strength of the aluminium, respectively. The yield strength of the weld for the L2 layer was between 51% and 52% of the yield strength of AA6061-T6 aluminium. The average values are shown graphically in Figure 18. 

The significantly lower tensile strength of the entire joint compared to the tensile strength of the specimens from the L2 layer was caused, among other things, by insufficient welding of the materials on the root side, as discussed previously (Figure 11). This resulted in eccentric loading of specimens during the monotonic tensile test and tearing of the joints. The extent of the non-welded section decreases with the increase offset value (Figure 11). However, there were some differences in the joint strength even for the highest offset value, for which the material microstructure across the joint thickness was characterised by a similar structure. 

**Figure 19:**
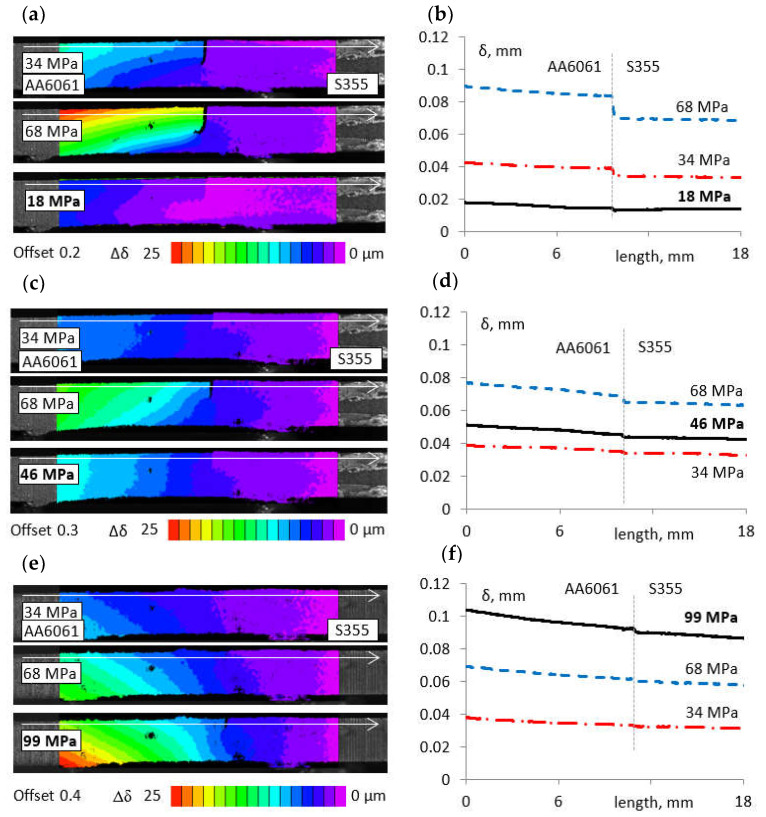
Examples of displacement distributions Δδ in the phase of joint integrity loss in the loading direction for specimens with 0.2 mm (**a**), 0.3 mm (**c**), and 0.4 mm (**e**) offsets; displacement distributions δ in the joint integrity loss phase in the selected cross sections of joints made with offsets of 0.2 mm (**b**), 0.3 mm (**d**), and 0.4 mm (**f**).

In order to illustrate the failure pattern of the joint, an analysis of the displacement distributions in the specimen during the monotonically variable tensile test was carried out. Figure 19a,c,e show example extents of displacement distributions Δδ in the load direction for specimens with 0.2, 0.3, and 0.4 mm offsets in selected loading phases: 34 and 68 MPa. Moreover, Figure 19b,d,f show displacement distributions δ in the phase of the joint integrity loss for the selected joint cross section indicated by the white axis in the figure. The lack of joint integrity causes a step change in displacement. By analysing the pattern of displacement changes along the specimen near the joint root (along the cross section marked with a white line in Figure 19a,c,e), the approximate value of the nominal stress S at which the joint lost its integrity in the analysed cross section was determined. For comparison purposes, the loss of integrity was assumed to occur when there was at least a 1 μm increase in the displacement in the transition line between the steel and aluminium alloy.

For specimens prepared using an offset of 0.2 mm, the discontinuity of the displacement occurred practically from the smallest load values, but the assumed displacement change/step was observed for S = 18 MPa. The stress value in the joint made using an offset of 0.3 mm was 46 MPa, while for an offset of 0.4, it reached 99 MPa. 

Comparison of the determined nominal stress values, for which a clear loss of joint integration was observed near the root, confirmed the occurrence of a significantly higher strength of the joints made with an offset value of 0.4 mm. However, also in this case, joint failure starts from the root and causes eccentric tearing of the joint.

It should be noted that the difference in joint strength along the weld line can have an adverse effect particularly in the case of loads that are variable over time.

The experimental values of the ultimate strength σ_u_ and yield strength σ_y_ were compared with their values determined theoretically based on the results of microhardness measurements.

The relationships used to determine the mechanical properties based on the HV hardness of aluminium alloys have been adapted from [40]:

Model 1:

(1) $$\sigma_{u}(MPa)=2.4079\;HV+46.39$$

(2) $$\sigma_{y}(MPa)=2.9263\;HV-44.289$$

Model 2:

(3) $$\sigma_{u}(MPa)=2.4079\;HV+46.39$$

(4) $$\sigma_{y}\left(MPa\right)=2.11\;HV+0.0043\;{HV}^{2}-7.9$$

Taking into account the recrystallization effect of the aluminium alloy with the simultaneous strong reduction of grain size, their values were additionally determined according to the values of the correlation coefficients given in [41]:

Model 3:

(5) $$\sigma_{y}\left(\mathrm{MPa}\right)=2.49\;HV$$

(6) $$\sigma_{u}\left(\mathrm{MPa}\right)=3.01\;HV$$

The properties designated for the selected connection zone are collected in Table 6 and Table 7.

The yield strength and ultimate tensile strength values determined for the WNZ based on the microhardness measurements are significantly lower than the experimental values, irrespective of the model used, and they range from 28.2 to 8.2%. The situation is reversed for the base material, with the calculated yield strength and ultimate tensile strength values being much closer to the experimental values, and the differences ranging from 2.5 to 11%.

The AA6061-T6 aluminium is a supersaturated and artificially aged alloy which significantly improves its mechanical properties such as tensile strength and hardness. During FSW, the materials subject to joining soften and mix, which causes a decrease in these properties. The FSW joint was analysed on the basis of the welded joint strength requirements for the AA6061-T6 aluminium alloy. The International Association of Classification Societies (IACS) specifies the minimum tensile strength for the welded joint of the AA6061-T6 aluminium alloy at 170 MPa [42]. The authors in [43] specify the minimum tensile strength for a welded joint of the AA6061-T6 aluminium alloy at 160 MPa and the offset yield point R_P0.2_ at 125 MPa. The confidence intervals were determined for the average value of R_m_ and R_P0.2_ with the statistical significance of a = 0.1. The values of the intervals are shown in Table 8.

The S355/AA6061-T6 FSW joint made with 0.3 mm and 0.4 mm offsets met both of the strength criteria set. The joint made with an offset of 0.2 mm did not meet the minimum tensile strength requirements.

### 3.4. Fractography

**Figure 20:**
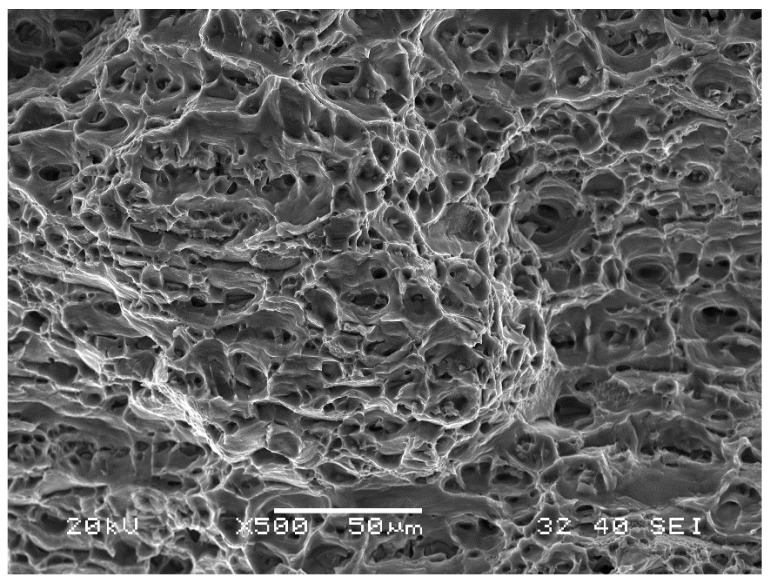
The cross section of a W1.1 specimen.

Figure 20 shows an example of a fractured surface of a W1.1 aluminium specimen. The uniform distribution of the dimples clearly indicates the ductile nature of the fracture, typical for the analysed AA6061-T6 alloy.

**Figure 21:**
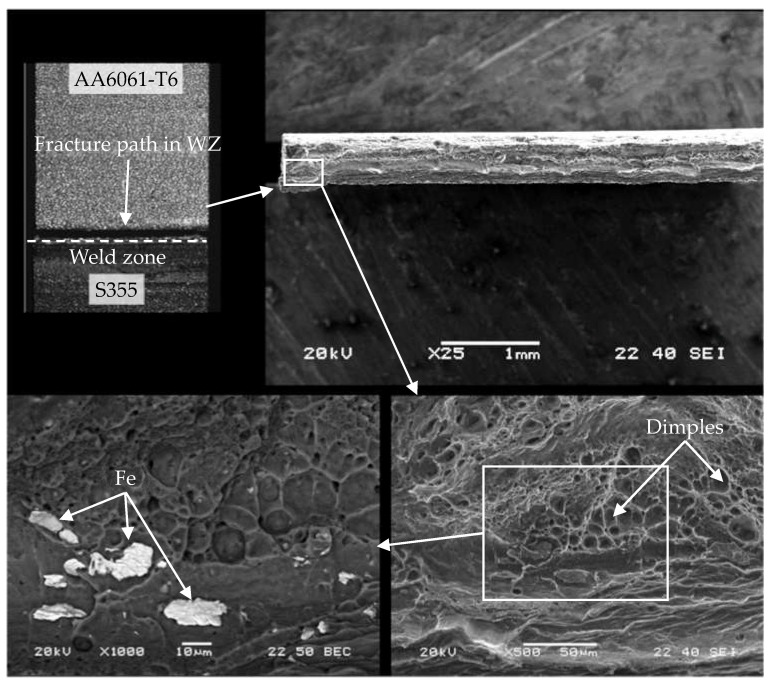
Cross section of WM1.0 specimen made with an offset of 0.2 mm.

The WM1.0 specimens prepared with an offset of 0.2 mm (Figure 21) fractured during the monotonic tensile test in the interface area between the aluminium alloy and steel. The fracture was mixed in nature with a predominance of dimples of various sizes characteristic of a ductile fracture. Steel inclusions formed during the FSW process caused a smaller cross-section surface reduction in the specimen’s fracture area. The presence of steel inclusions changed the nature of the fracture from ductile to brittle.

**Figure 22:**
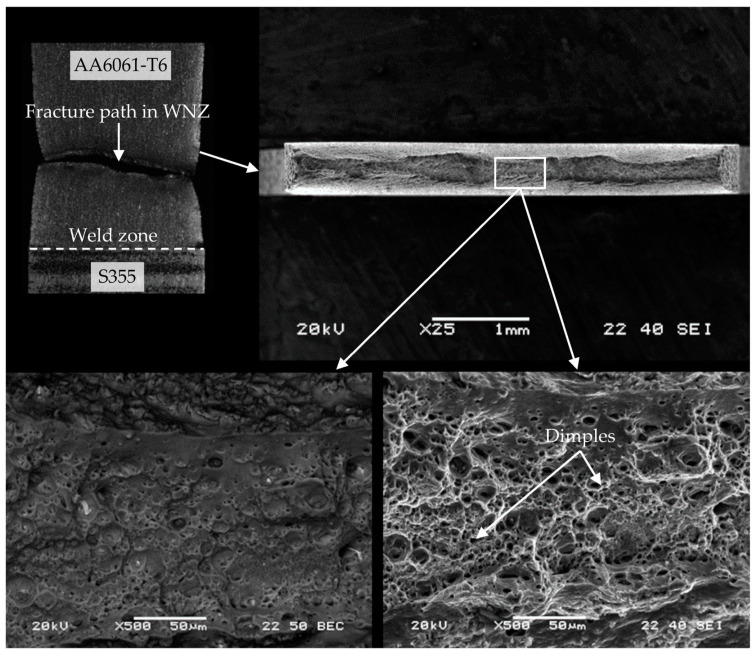
The cross section of WM1.0 specimen made with an offset of 0.3 mm.

Fracturing of WM1.0 specimens prepared with an offset of 0.3 mm during the monotonic tensile test occurred mainly in the mixed WNZ zone. The fracture was ductile with no visible steel inclusions (Figure 22). A characteristic feature of the fractures of joints made with an 0.3 mm offset was a significant reduction of the specimen thickness during the monotonic tensile test.

**Figure 23:**
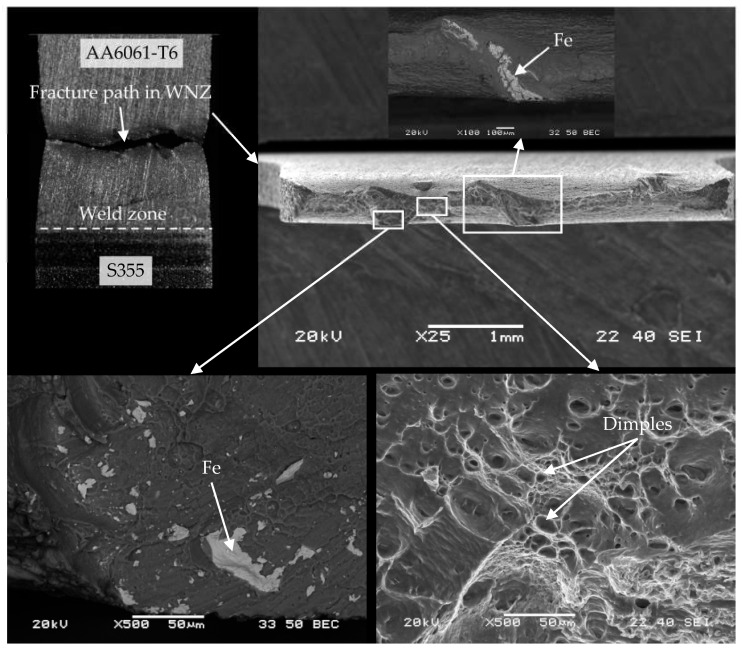
The cross section of WM1.0 specimen made with an offset of 0.4 mm.

Fracturing of WM1.0 specimens prepared with an offset of 0.4 mm during the monotonic tensile test occurred mainly in the mixed WNZ zone. The fracture was mixed in nature with a predominance of dimples of various sizes characteristic of a ductile fracture (Figure 23). The characteristic features of the joint fracture for an offset of 0.4 mm consisted of fine and large steel inclusions formed during the FSW process, which changed the character of the fracture, and a significant reduction of the specimen’s thickness during the monotonic tensile test only in areas where no steel inclusions were observed. The much higher standard deviation value of the mean tensile strength of the joint made with a 0.4 mm offset than with a 0.3 mm offset resulted from steel inclusions of different sizes, which were also observed during microstructural analysis.

## 4. Conclusions

The subject of the tests discussed in this paper were S355/AA6061-T6 joints made by friction stir welding (FSW). The following conclusions can be drawn on the basis of the analysis of the research carried out, including the study of the effect of selected process parameters on the tensile strength of macro and microspecimens of the joint, with fractography, a description of the macro and microstructure of the joint, and the microhardness tests in the cross section of the joint.

- Hardness measurements along the cross section of the joint confirmed the weakening of the aluminium alloy in the weld zone regardless of the offset value used, and showed an increase in the steel hardness near the joint proportional to the value of the offset parameter. The analysis of the mechanical properties using hardness makes it possible to determine approximate values of the ultimate strength of the aluminium alloy in the weld zone with an inaccuracy of about 10%. However, in the case of yield strength, the inaccuracy increases to about 30%.
- The S355/AA6061-T6 joint in the L2 layer (WM1.0 minispecimens), made using offsets of 0.3 mm and 0.4 mm, meets the criteria for weld joints carried out using conventional methods, although the lack of a correct joint in the weld root needs to be resolved for the entire joint to be applicable.
- The entire S355/AA6061-T6 FSW joint (W2.0 macrospecimens) was characterized by the maximum average tensile strength of 141 MPa for an offset value of 0.4 mm, which was 45% of the strength of the AA6061-T6 parent material.
- The S355/AA6061-T6 FSW joint in the L2 layer was characterised by the tensile strength of 185 MPa for an offset value of 0.3 mm and 182 MPa for an offset value of 0.4 mm. The difference between the strength of the entire joint compared to the strength of the joint in the L2 layer is due to the insufficient integrity of the joint in the root zone.
- Fracturing of the specimens of the joint taken from the L2 layer during the monotonic tensile test occurred at the steel-aluminium alloy interface for the 0.2 mm offset and in the WNZ mixing zone for the 0.3 mm and 0.4 mm offsets, where the lowest hardness was recorded.
- Fractures of WM1.0 specimens taken from the L2 layer, for each offset value, were mixed in character with a predominance of dimples of various sizes characteristic of a ductile fracture. More numerous and larger base steel inclusions were observed in the fractures of WM1.0 specimens for the 0.4 mm offset compared to the other offset values.

## Figures and Tables

**Table 1:** Chemical composition of AA6061-T6 aluminium alloy, wt.%.

Al	Si	Fe	Cu	Mn	Mg	Cr	Zn	Ti
97.51	0.62	0.4	0.18	0.1	1	0.07	0.05	0.07

**Table 2:** Chemical composition of S355 steel, wt.%.

Fe	C	Mn	Si	P	S	Cu
97.05	0.2	1.6	0.55	0.025	0.025	0.55

**Table 3:** Process parameters of the FSW joint.

Process Parameters			
Name	Plate 0.2	Plate 0.3	Plate 0.4
Offset	0.2 mm	0.3 mm	0.4 mm
Rotational speed	250 RPM		
Welding velocity	2.5 cm/min		
Detention time	0.5 s		
Pin height	2.8 mm		
Pin diameter	5 mm		
Tool inclination	2°		
Tool penetration depth	2.9 mm		

**Table 4:** Average tensile strength of base materials and joint types analysed.

Type	Base Materials		FSW Joint W2.0		
	AA6061-T6	S355	Offset 0.2	Offset 0.3	Offset 0.4
s_u_, MPa	312 ± 0.8	506 ± 1.4	116 ± 3.6	125 ± 5.5	141 ± 4.6

**Table 5:** Average tensile strength, yield strength, and Young’s modulus of the base materials and layer L2 of the joint types analysed.

Type	Base Materials		FSW Joint WM1.0		
	AA6061-T6	S355	Offset 0.2	Offset 0.3	Offset 0.4
s_u_, MPa	312 + 0.8	506 + 1.4	159 + 10	185 + 8	182 + 9.3
s_y_, MPa	279 + 1	479 + 16.7	145 + 7.6	145 + 3.9	142 + 12.8
E, MPa	72,762 + 1254	224,083 + 5356	-	-	-
E *, MPa	-	-	64,990 + 850	66,734 + 3816	67,012 + 3607

E *—Young’s moduli were calculated from the strains determined using DIC.

**Table 6:** Comparison of tensile and yield strength values determined by numerical and experimental methods for BM.

Model	BM			Experimental		BM	
	HV	*σ_u_* MPa	*σ_y_* MPa	*σ_u_* MPa	*σ_y_* MPa	Δ*σ_u_* %	Δ*σ_y_* %
Model 1	115	323	292	312	279	3.5	4.7
Model 2		323	292			3.5	4.7
Model 3		346	286			10.9	2.5
$∆\sigma_{u\;BM}=\frac{\sigma_{u\;BM}-\sigma_{u\;exp}}{\sigma_{u\;exp}}\times 100\%,\;∆\sigma_{y\;BM}=\frac{\sigma_{y\;BM}-\sigma_{y\;exp}}{\sigma_{y\;exp}}\times 100\%$							

**Table 7:** Comparison of tensile and yield strength values determined by numerical and experimental methods for WNZ.

Model	WNZ			Experimental		WNZ	
	HV	*σ_u_* MPa	*σ_y_* MPa	*σ_u_* MPa	*σ_y_* MPa	Δ*σ_u_* %	Δ*σ_y_* %
Model 1	50	167	102	182	142	−8.2	−28.2
Model 2		167	108			−8.2	−23.9
Model 3		151	125			−17.0	−12.0
$∆\sigma_{u\;WNZ}=\frac{\sigma_{u\;WNZ}-\sigma_{u\;exp}}{\sigma_{u\;exp}}\times 100\%,\;∆\sigma_{y\;WNZ}=\frac{\sigma_{y\;WNZ}-\sigma_{y\;exp}}{\sigma_{y\;exp}}\times 100\%$							

**Table 8:** Confidence intervals for the average value.

Type	0.2	0.3	0.4
R_m_, MPa	150.2 < d < 166.9	178.2 < d < 191.5	174.7 < d < 190
R_P0.2_, MPa	139.1 < d < 151.5	141.8 < d < 148.1	131.8 < d < 152.8

## Data Availability

The raw/processed data required to reproduce these findings cannot be shared at this time due to technical or time limitations.
